# Recent Advances in Superhydrophobic Electrodeposits

**DOI:** 10.3390/ma9030151

**Published:** 2016-03-03

**Authors:** Jason Tam, Gino Palumbo, Uwe Erb

**Affiliations:** 1 Department of Materials Science and Engineering, University of Toronto, 184 College Street, Toronto, ON M5S 3E4, Canada; jht.tam@mail.utoronto.ca; 2 Integran Technologies Inc., 6300 Northam Drive, Mississauga, ON L4V 1H7, Canada; Palumbo@integran.com

**Keywords:** superhydrophobicity, non-wetting metal surfaces, electrodeposition, surface engineering

## Abstract

In this review, we present an extensive summary of research on superhydrophobic electrodeposits reported in the literature over the past decade. As a synthesis technique, electrodeposition is a simple and scalable process to produce non-wetting metal surfaces. There are three main categories of superhydrophobic surfaces made by electrodeposition: (i) electrodeposits that are inherently non-wetting due to hierarchical roughness generated from the process; (ii) electrodeposits with plated surface roughness that are further modified with low surface energy material; (iii) composite electrodeposits with co-deposited inert and hydrophobic particles. A recently developed strategy to improve the durability during the application of superhydrophobic electrodeposits by controlling the microstructure of the metal matrix and the co-deposition of hydrophobic ceramic particles will also be addressed.

## 1. Introduction

Superhydrophobic surfaces are highly water repellent surfaces with a water contact angle (WCA) >150° and a very low water sliding angle (SA) [1]. They were first observed on plants and animals, with the lotus leaf being the most widely-known example. The lotus leaf achieves its non-wetting property by a combination of specific chemical and microstructural characteristics: nanoscale hydrophobic epicuticular wax crystals superimposed on microscale papillae [2]. Due to its high water repellency, the lotus leaf also has a self-cleaning mechanism, known as the lotus-effect, where contaminating particles on the leaf surface can be easily removed by rain water droplets [2]. This phenomenon observed in nature has generated considerable research activity over the past decade to reproduce superhydrophobicity on engineering materials for practical applications, such as self-cleaning, anti-icing, anti-fouling, anti-corrosion and reduced fluid drag surfaces [3,4,5,6,7,8,9]. There are many methods reported in the literature to produce superhydrophobic surfaces, such as lithography [10,11,12,13], templating [3,14,15], femtosecond laser pulsing [16,17], etching [18,19,20], sol-gel techniques [21,22], thermal chemical vapor deposition [23] and electrochemical processes [24]. However, some of these techniques, for instance lithography and femtosecond laser pulsing, are very expensive and difficult to scale up for large structures. Although templating techniques have great potential for large-scale production, they are often limited to soft polymers only (e.g., [3,14]). On the other hand, electrodeposition is a promising approach to fabricate superhydrophobic surfaces due to its simplicity, low cost and ease of scalability. In addition, electrodeposits can be applied on a wide range of materials, including metals, composites and polymers [25].

The purpose of this review article is to provide an overview of progress made in the area of superhydrophobic surfaces prepared by electrodeposition over the past decade. First, some fundamental aspects of wetting behavior used in the context of this review will be summarized. Then a comprehensive review on superhydrophobic surfaces made by electrodeposition to date will be presented. This will be followed by a discussion of more recent developments, the mechanical and wear stability of such structures and future application potentials and commercialization challenges for this type of non-wetting metallic surfaces.

## 2. Wetting Behavior

**Figure 1:**
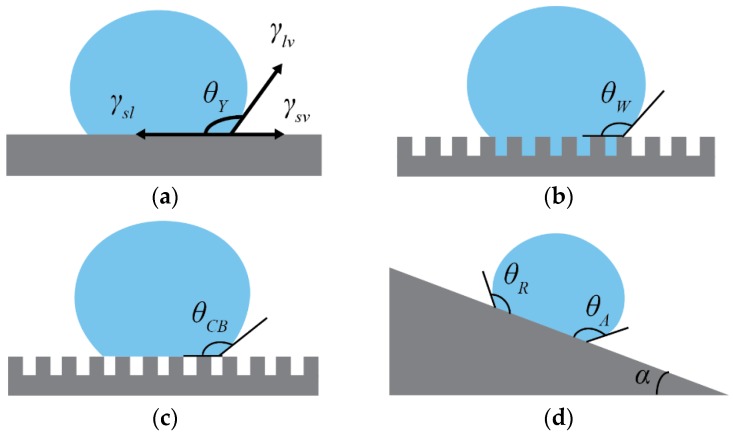
(**a**) Young’s equation; (**b**) Wenzel state; (**c**) Cassie–Baxter state; (**d**) water droplet on an incline, showing advancing and receding contact angle and sliding angle.

The fundamental equation that describes a liquid droplet at rest on an ideal, smooth surface is Young’s equation, given by:

(1) $$\gamma_{sv}=\gamma_{lv}\cos\theta_{Y}+\gamma_{sl}$$

where γ*_sv_*, γ*_lv_*, γ*_sl_* are the surface tensions for solid-vapour, liquid-vapour and solid-liquid interfaces, respectively, and θ*_Y_* is the contact angle of the liquid droplet (Figure 1a). For water, when the contact angle is less than 90°, the surface is said to be hydrophilic. Between 90° and 150°, the surface is hydrophobic. If the water contact angle is greater than 150°, the surface is defined to be superhydrophobic. However, the largest water contact angle θ*_Y_* reported for smooth solid surfaces is about 120° [26]. In order to achieve superhydrophobicity, surface roughness is also required.

There are two main theories that describe the wetting of rough surfaces, namely the Wenzel and Cassie–Baxter models. A schematic diagram of a liquid droplet on a rough surface, according to the Wenzel model, is shown in Figure 1b. The Wenzel model describes homogeneous wetting, where the liquid droplet is in contact with the peaks and valleys of the rough surface [27] with the following equation:

(2) $$\cos\theta_{W}=R_{f}\cos\theta_{Y}$$

where θ*_W_* is the Wenzel contact angle and *R_f_* is the roughness factor, which is defined as the surface area ratio between the rough surface and its projection on a 2D plane. For a surface that is intrinsically hydrophilic, θ*_Y_* < 90°, an increasing roughness factor renders the surface more hydrophilic, *i.e.*, a decrease in θ*_W_* is observed. Similarly, hydrophobicity can be enhanced with increased roughness for a surface that is intrinsically hydrophobic. However, increases in roughness also increases the solid-liquid interface; hence, the surface in the Wenzel state is “sticky”.

The Cassie–Baxter model describes heterogeneous wetting, where the liquid droplet is suspended by the peaks and does not penetrate into the valleys filled with air [28], as shown in Figure 1c. The Cassie–Baxter equation is derived from Cassie’s law, which describes wetting for a two-component surface [29]:

(3) $$\cos\theta_{C}=f_{1}\cos\theta_{1}+f_{2}\cos\theta_{2}$$

where *f*_1_ and *f*_2_ are the area fractions of Components 1 and 2, respectively, θ_1_ and θ_2_ are the contact angles of Components 1 and 2, respectively, and θ*_C_* is the apparent contact angle of the composite. Air trapped in the valleys between the solid and liquid can be treated as one component with a water/air contact angle of 180°. Equation (3) can then be rewritten to form the Cassie–Baxter equation [28]:

(4) $$\cos\theta_{CB}=f_{1}(\cos\theta_{1}+1)-1$$

where *f*_1_ is the area fraction of the solid surface in contact with the liquid droplet and θ*_CB_* is the contact angle of the liquid droplet according to Cassie–Baxter. In this case, increasing the area fraction of air 1 − *f*_1_ will increase the contact angle and reduce the adhesion of the liquid droplet to the surface; thus, the surface is “slippery”. Furthermore, even if a material is intrinsically hydrophilic, it can be modified to become hydrophobic by increasing the surface roughness to introduce trapped air pockets.

An irreversible transition from the Cassie–Baxter to the Wenzel state is observed for many surfaces, where the composite interface of trapped air pockets in the valleys of the rough surface is destroyed, and the valleys are filled with the liquid to form a homogenous solid-liquid interface and a decrease in the apparent contact angle [30]. The transition from the Cassie–Baxter to the Wenzel state can be caused by many factors, including chemical and surface roughness inhomogeneity, geometry and the profile of surface roughness [30].

Adhesion of a liquid droplet to a surface can be related to the contact angle hysteresis (CAH), Δθ. Contact angle hysteresis is the difference between the advancing contact angle θ*_A_* and the receding contact angle θ*_R_*. For a liquid droplet on an inclined surface, the advancing contact angle is the contact angle on the lower side (advancing side), and the receding contact angle is the contact angle on the upper side (receding side) just before the droplet slides off when the incline reaches a critical angle, known as the sliding or roll off angle α. A schematic diagram of a liquid droplet on an inclined surface, showing contact angle hysteresis, is presented in Figure 1d. Contact angle hysteresis can be related to the sliding angle by the following equation [31]:

(5) $$mg\sin\alpha =w\gamma_{lv}(\cos\theta_{R}-\cos\theta_{A})$$

where *g* is the force due to gravity and *m* and *w* are the mass and width of the droplet, respectively. According to this equation, the sliding angle α is minimized when the contact angle hysteresis Δθ is small. Surface roughness also has a significant effect on the contact angle hysteresis. For instance, when the surface is in the Wenzel wetting state, the liquid droplet will remain on the surface even with a high tilt angle due to surface roughness, providing pinning points for the liquid droplet [32]. On the other hand, when the rough surface is in the Cassie–Baxter wetting state, a droplet will roll off the surface with a small tilt angle due to the low area fraction of the solid-liquid interface and, hence, the high area fraction of the liquid-air interface. In addition to the high contact angle, the sliding angle should be less than 10° for superhydrophobic surfaces [1]. When the sliding angle is low, the liquid droplet has low adhesion, and the self-cleaning effect is achieved; water droplets will easily roll off the surface and will carry dirt and contaminants along the way.

As noted earlier, natural superhydrophobic surfaces, such as the lotus leaf or the legs of the water strider, have hierarchical nanoscale and microscale roughness. The exact reason why natural superhydrophobic surfaces exhibit hierarchical roughness is not well understood. According to Neinhuis and Barthlott’s study [33] on water-repellent leaves, the effect of mechanical abrasion on water repellency is minimized on leaves with hierarchical roughness, as only the tips of the papillae would be affected by wear, and the nanostructured wax crystals in the valleys would be protected. Thus, the water-repellent properties are not strongly affected. Nosonovsky and Bhushan [34] carried out a theoretical and experimental study on the effect of hierarchical roughness on contact angle and contact angle hysteresis. They identified some of the mechanisms that can cause the destabilization of the liquid-air-solid interface in the Cassie–Baxter wetting state, including capillary waves, condensation and accumulation of nanodroplets, and surface inhomogeneity. Since these destabilization mechanisms are scale dependent, each with different characteristic scale lengths, hierarchical, multi-scale roughness is required to resist the destruction of the liquid-air-solid interface.

## 3. Superhydrophobic Surfaces by Electrodeposition

**Figure 2:**
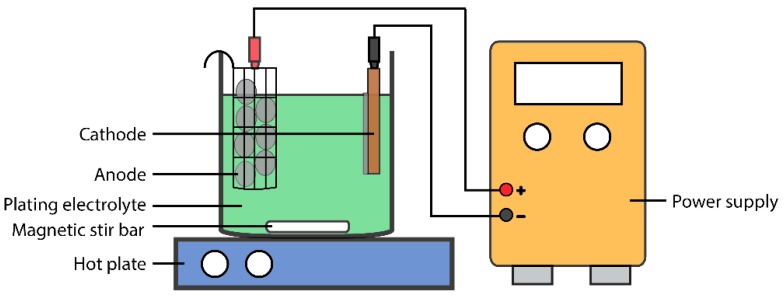
Schematic diagram of a lab-scale electroplating setup.

Electrodeposition is a widely-used metal deposition technology to coat the underlying metal substrate for improvements in appearance, wear resistance and corrosion resistance [25]. Typically, an electrodeposition setup consists of an anode and a cathode (usually the substrate to be coated) immersed in an electrolyte containing metal ions, typically in aqueous, organic or ionic liquid solutions. An electrical potential is applied between the two electrodes to dissolve metal into metal ions at the anode and reduce metal ions at the cathode to form a metal coating. A schematic diagram of a typical lab-scale electrodeposition setup is shown in Figure 2. The surface morphology of the electrodeposit can be controlled by a variety of parameters, such as current density, the addition of bath additives and bath chemistry.

**Figure 3:**
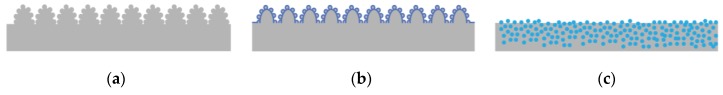
Cross-sectional schematic diagrams of: (**a**) an intrinsically rough superhydrophobic surface; (**b**) a rough surface modified by low surface energy material to achieve superhydrophobicity; (**c**) metal matrix composite with hydrophobic particles.

Superhydrophobic surfaces made by electrodeposition can be classified into three main categories, as shown in Figure 3 and Table 1. Superhydrophobicity is achieved by: (a) surface roughness alone; (b) surface roughness and surface chemical modification with low surface energy material; and (c) co-deposition of hydrophobic particles with a metal matrix. Cross-sectional views of the three types of electrodeposits are shown in Figure 3. It should be noted that there has also been significant work on co-depositing hydrophobic particles by the electroless deposition process (e.g., [35,36,37,38,39]). However, these studies will not be included in this review, because the electroless deposition process is quite different.

### 3.1. Surface Roughness-Based Superhydrophobic Electrodeposits

In the following sections, superhydrophobic electrodeposits, without post-deposition chemical modifications by low surface energy material, are reviewed (Table 1, Electrodeposit Category a). The synthesis processes of such electrodeposits are usually simple and easy to scale up.

#### 3.1.1. Nickel

Due to its outstanding mechanical and corrosion properties, nickel is a good candidate material to produce superhydrophobic surfaces. As shown in Table 1, Electrodeposit Category a, superhydrophobic nickel surfaces have been produced from aqueous, ionic and organic electrolytes.

Hang *et al.* [40] first reported superhydrophobic Ni surfaces produced from aqueous solution containing ethylenediamine dihydrochloride, an additive that promotes the formation of cone-shaped Ni crystals. The electrodeposition process involved two steps; microcone arrays were first deposited at a current density of 20 mA/cm^2^ for 10 min. Subsequently, nanocone arrays were deposited onto the surface of the microcone arrays at a higher current density of 50 mA/cm^2^ to form a hierarchical micro-nano-surface roughness. The water contact angle of the as-deposited surface was 154°, which was explained by the Cassie–Baxter wetting state, as the dual scale roughness allowed large fractions of trapped air pockets for a high contact angle on an intrinsically hydrophilic material. The contact angle of water on a smooth electrodeposited Ni surface is between 79° and 87° [40,41]. Khorsand *et al.* [41] reported a similar study and achieved water contact angles up to 155°. The corrosion resistance of the superhydrophobic coatings was measured by electrochemical impedance spectroscopy and Tafel polarization. The authors determined that the corrosion rate of the superhydrophobic hierarchical cone array surface was only 1% of the rate for the smooth nickel surface. Such coatings can be potentially employed in corrosive environments, such as marine applications.

**Figure 4:**
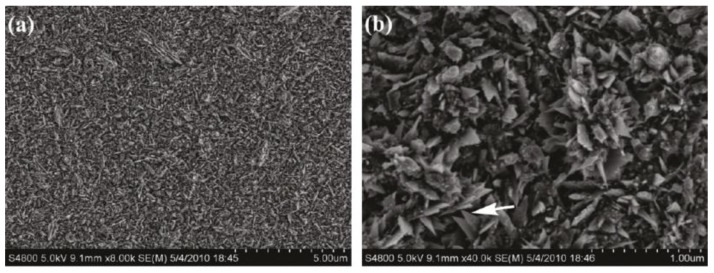
Low magnification (**a**) and high magnification (**b**) SEM images of a Ni film with nanosheet morphology formed by the constant voltage (CV) mode from an ionic electrolyte. Reprinted (adapted) with permission from [42]. Copyright 2011, American Chemical Society.

Superhydrophobic Ni films with different surface morphologies were also produced using an ionic electrolyte with varying plating waveforms. As reported by Gu and Tu [42], the ionic electrolyte was a deep eutectic solvent composed of choline chloride, ethylene glycol and nickel chloride. Compared to aqueous electrolyte, ionic electrolyte has a very low vapor pressure and high thermal stability, which allow for greater control options during the synthesis process [42]. In this study, the electrodeposition process was carried out at 90 °C with three different voltage waveforms: constant voltage (CV), pulse voltage (PV) and reverse pulse voltage (RPV). Each voltage waveform resulted in different surface morphologies and roughness of the Ni film. In the CV mode, the surface of the Ni film consisted of nanosheets, as shown in Figure 4. When the voltage waveform was PV, aligned nanostrips were formed. A hierarchical flower-like structure was the resulting morphology from the RPV mode. The Ni films produced by the three voltage waveform were all hydrophobic. Without any chemical modification, contact angles up to 164° and sliding angles less than 3° were reported for the Ni film with the nanosheet morphology. In addition, corrosion measurements have shown that the superhydrophobic Ni films have a lower corrosion potential than the brass substrate, making them suitable as a corrosion barrier for applications in aqueous environments.

The Ni with a cauliflower surface morphology was produced from an organic electrolyte containing ethanol, nickel chloride and myristic acid; a saturated fatty acid [43]. The as-prepared surface had excellent non-wetting properties: a water contact angle of 164° and a sliding angle less than 2°. The resulting cauliflower-like surface was composed of nickel and nickel myristate. As the Ni^2+^ ions were reduced to solid Ni at the cathode, nickel myristate was formed and simultaneously deposited when the Ni^2+^ ions near the cathode reacted with myristic acid in the presence of the applied potential.

#### 3.1.2. Copper

**Figure 5:**
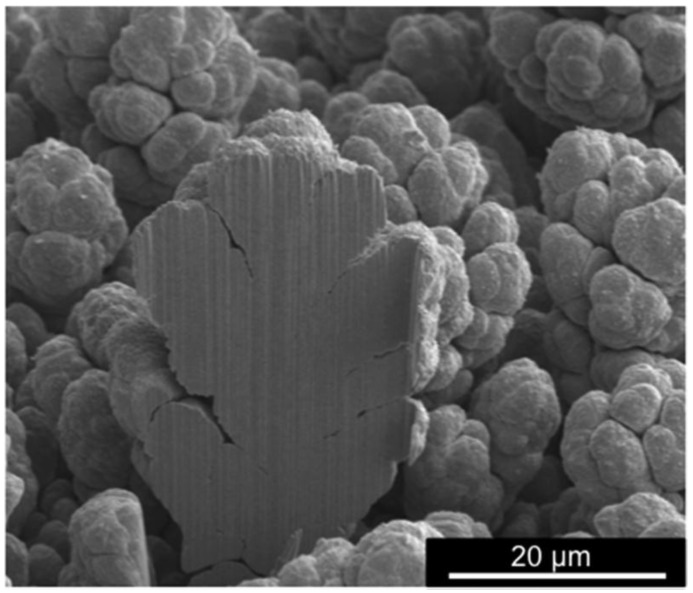
SEM image showing the cauliflower-like structure of Cu obtained by the two-step electrodeposition process. A focused ion beam was used to cut a cross-section to reveal the branches under the surface. Reprinted (adapted) with permission from [45]. Copyright 2014, American Chemical Society.

Superhydrophobic copper surfaces produced from an aqueous electrolyte without chemical modification were studied by Xi *et al.* [44] and Haghdoost and Pitchumani [45]. In Xi *et al.*’s study, Cu with a lotus leaf-like surface was produced by a one-step direct current plating process. The surface was superhydrophobic with a water contact angle of 153° and a sliding angle of 8° [44]. Unlike Xi *et al.*’s study, the electrodeposition process developed by Haghdoost and Pitchumani involved two steps [45]. First, a cauliflower-type structure was deposited by applying a voltage of 1.1 V. However, with increasing deposition time, some branches of the cauliflower structure become unstable and loosely attached to the surface. The loosely-attached structure was reattached to the deposit by applying a lower potential of 0.15 V for 10 s. The resulting surface morphology was a cauliflower-like structure (Figure 5), where the microscale branches were covered with sub-micron globular asperities. Due to the multi-scale roughness, the measured water contact angle and contact angle hysteresis of the surface were 160° and 5°, respectively.

Superhydrophobic copper surfaces with a spiky, flower-like morphology were produced with organic electrolyte containing ethanol and either fatty acid or organic acid, myristic acid or nonadecafluorodecanoic acid [46]. For the copper surface produced with the electrolyte containing myristic acid, copper myristate was formed along with copper. The water contact angle was 154°. In addition, the contact angle remained above 150° in salt water, as well as acidic and basic environments ranging from pH 0.3–13.8. When the electrolyte contained nonadecafluorodecanoic acid, the copper surface was superamphiphobic: contact angles of water and oil were 161° and 143°, respectively. Such surfaces could have a wide range of industrial applications, ranging from self-cleaning metal structures to oil pipelines for low fluid drag and anti-fouling.

#### 3.1.3. Cobalt

Using an aqueous electrolyte containing CoCl_2_ and Na_2_SO_4_, cobalt surfaces with colonies of flower-like structures were produced [47]. The as-deposited structure without modification had a water contact angle of 162° and low contact angle hysteresis of 3.5°.

Cobalt surfaces with a hierarchical micro-nano-scale globular structure were made with an organic electrolyte containing ethanol, myristic acid and CoCl_2_ [48]. The surfaces were composed of Co and cobalt myristate, a compound that formed during the electrodeposition process when Co^2+^ ions react with myristic acid near the cathode. The water contact angle of the as-deposited surfaces was 164°, and the sliding angle was less than 2°. The coatings remained stable in non-wetting properties, as it was determined that no obvious water contact angle change was observed after exposing the deposit to air for one year. In a study involving a similar plating electrolyte, but at lower applied potential, the resulting superhydrophobic Co coatings consisted of a micro-nanofiber structure, and a water contact angle of 160° and sliding angle of 6° were observed [49]. The coating also demonstrated excellent stability in a wide range of pH values. Between pH 3.0 and 11.0, the contact angles of the coating were greater than 150°, and the sliding angle remained less than 10° for a pH between 5.0 and 11.0.

#### 3.1.4. Zinc

A two-step process to produce superhydrophobic zinc coatings was developed by He *et al.* [50]. In this study, zinc coatings were first grown on copper substrate by electrodeposition in aqueous solution, followed by annealing at 190 °C. The annealing process transformed the as-deposited morphology from scaly sheets to willow leaf-like structures with submicron features. Furthermore, the main component of the surface after annealing was zinc oxide. Before annealing, the surface was hydrophilic (WCA = 15°). After annealing, the coatings demonstrated highly non-wetting properties with a water contact angle of 170° and a sliding angle of less than 1°.

#### 3.1.5. Bismuth

Bismuth surfaces with a porous dendritic morphology were produced by a one-step electrodeposition process using an aqueous solution as the electrolyte [51]. The effects of applied potential, deposition time and concentration of the electrolyte on wetting properties were studied. X-ray photoelectron spectroscopy (XPS) showed that the dendritic Bi surface was covered with a thin layer of Bi_2_O_3_ from the self-passivation of Bi. Oxides usually have a lower surface energy than the metal, which contributes to producing non-wetting surfaces. This was determined when the applied potential was −1.8 V *vs.* the saturated mercurous sulfate electrode, and for an electrodeposition time of 30 s, a maximum water contact angle of 164° was achieved.

#### 3.1.6. Manganese

A manganese surface with a cauliflower-like morphology was fabricated by electrodeposition with an organic solution of ethanol, MgCl_2_ and myristic acid [52]. Water contact angles up to 163° and sliding angles less than 3° were reported. XRD and FTIR analysis showed that low surface energy manganese myristate was formed on the superhydrophobic surface.

#### 3.1.7. Lanthanum and Cerium

Lanthanum and cerium surfaces with spiky and micro-nano-scale papillae morphologies were electrodeposited with organic electrolyte comprised of ethanol, myristic acid and the corresponding lanthanide salt [53,54,55]. The effect of electrodeposition time on surface morphology and wetting properties was explored. In all studies, when the deposition time was short, only small particles were formed on the surface. With increasing plating time, the particles evolved into a spiky, flower-like structure with nanorods and a papillae-like morphology with dual scale roughness, which promotes non-wetting behavior. Water contact angles were above 160°. Similar to other studies involving organic electrolytes with myristic acid, the surface of the superhydrophobic metal also contained metal myristate. In addition, when the superhydrophobic cerium coating was applied on a magnesium alloy substrate, improved corrosion properties were observed [54,55]. The authors suggested that the technique is a fast and simple process to protect magnesium alloys and may open up new applications for these lightweight materials.

#### 3.1.8. Ni-Cu-P Alloy

In addition to pure metals, alloys with non-wetting properties can also be fabricated by electrodeposition. In Yu *et al.*’s study [56], superhydrophobic Ni-Cu-P alloy coatings were made from aqueous electrolyte. A hierarchical cauliflower-like surface morphology was observed on the surface of the alloy coating, while a pure nickel coating deposited under the same conditions was smooth. The cauliflower-like structure was formed on the alloy coating due to the difference in the reduction potentials of Ni and Cu. Cu would nucleate on the cathode first, leading to uneven current density on the surface. Next, Ni would deposit and grow preferentially on the Cu particles, contributing to surface roughness, since electrodeposition occurs faster on raised surfaces. The water contact angle of the alloy was 153°. Interestingly, despite the high contact angle, the water droplet remained adhered to the surface even when the surface was turned upside down, which suggests that the surface was in the sticky Wenzel state. Based on this phenomenon, the author proposed that the coating can be applied to no-loss micro-liquid droplet transportation.

### 3.2. Surface Roughness and Chemical Modification-Based Superhydrophobic Electrodeposits

In this approach, a rough metal surface is first synthesized by electrodeposition. Subsequently, the rough metal surface is rendered superhydrophobic by immersion/dipping treatment with low surface energy substances, such as saturated fatty acid or fluorinated solutions (Table 1, Electrodeposit Category b).

#### 3.2.1. Nickel

Electrodeposited nickel surfaces with different surface morphologies can be made superhydrophobic by low surface energy treatment [57,58,59]. Su and Yao [58] fabricated a Ni coating with a pine cone-like morphology in a two-step process: (i) electrodeposition in a Watts bath; and (ii) heat treatment in an oven with a fluorinated solution. After the treatment, water contact angles up to 162° and sliding angles of 3° were observed. In Chen *et al.*’s study [57], a nickel surface with nanocone arrays was deposited from an electrolyte containing NiCl_2_, H_3_BO_3_ and a crystal modifier. The effects of current density and plating time on the surface morphology and wetting behavior were studied. After electrodeposition, the coating was dipped in stearic acid, an 18-carbon chain saturated fatty acid. When the plating time and current density were 1 min and 10 mA/cm^2^, respectively, the average height of the nanocones was 214 nm, and the average root diameter was 90 nm. The water contact angle of the resulting Ni surface was 148°, but water droplets did not roll off when the surface was tilted. When the electrodeposition was performed at 20 mA/cm^2^ for 10 min, the height and root diameter of the nanocones increased to 872 nm and 500 nm, respectively. The water contact angle increased slightly to 155°, but more importantly, the sliding angle decreased to less than 1°. It was found that when the cone height was increased, water intrusion into the valleys between the cones was reduced, which suggests that water droplets are less likely to be pinned by the cone tips.

Superhydrophobic nickel film with a surface morphology similar to the lotus leaf was produced by a multi-step replica technique involving electrodeposition and treatment with a low surface energy chemical [59]. First, a negative impression of a lotus leaf was replicated on cellulose acetate film, followed by sputtering of gold onto the acetate film to obtain a conductive surface for subsequent electrodeposition. Nickel was then plated on the gold-coated acetate film, and the acetate film was dissolved in acetone to obtain a free-standing lotus leaf replica. Subsequently, a short secondary electrodeposition process was carried out to deposit spherical cups with smaller curvature on the tip of the protuberances. The surfaces were then submerged in perfluoropolyether solution to obtain water contact angles up to 156°.

#### 3.2.2. Copper

After treatment with low surface energy materials, electrodeposited copper with different surface morphologies can also be made superhydrophobic [6,60,61,62]. For instance, Wang *et al.* [61] electrodeposited copper with hierarchical spherical microstructure on indium tin oxide (ITO) substrates using a copper nitrate electrolyte and constant voltage of −0.25 V *vs.* a Ag/AgCl reference electrode. Following the deposition, the coating was immersed in n-dodecanethiol solution to make the surface superhydrophobic (WCA = 152°). A similar microstructure was also reported in a different study [62], where the coating was electrodeposited from a copper sulfate electrolyte. The coatings were then immersed in fluorocarbon emulsion, followed by a heat treatment process to obtain a water contact angle close to 160° and a contact angle hysteresis less than 2°.

In another study, Wang and colleagues [6] produced copper surfaces via a two-step electrodeposition process, followed by treatment with n-octanoic acid. In the first step of electrodeposition, a low overpotential of −0.8 to −1.0 V was applied to create nucleation sites on an ITO-coated glass substrate. In the second step of electrodeposition, a higher overpotential was applied to promote the growth of Cu particles on the substrate and to produce nanostructure protuberances. The final surface morphology of the electrodeposition process was nanoscale protrusions superimposed on microclusters. The water contact angle of the structured Cu surface was 153° after the treatment with n-octanoic acid.

#### 3.2.3. Zinc and Zinc Oxide

Electrodeposition of zinc can produce surfaces with spongy, fractal and nanorod morphologies [63,64,65]. Zhang *et al.* [63] produced zinc with a hierarchical spongy morphology by electrodeposition from ionic liquid. The formation of the spongy structure was due to the use of thiourea in the electrolyte. The thiourea molecules are adsorbed on the cathode and regulate the diffusion of zinc ions, causing a three-dimensional structure to form instead of a smooth film. After electrodeposition, the surface was immersed in a polypropylene solution to achieve a very high water contact angle of 170°.

Zinc films with fractal morphologies were electrodeposited onto steel substrates from an aqueous solution containing zinc salts [64]. The zinc surfaces were then sprayed with a room temperature vulcanized silicone polymer to change the wetting behavior from hydrophilic (WCA = 2°) to superhydrophobic (WCA = 155°, SA = 2°). Furthermore, the superhydrophobic zinc coatings showed improved corrosion resistance and reduced ice adhesion compared to the bare steel substrate. This type of coatings can be applied to equipment, buildings and infrastructures in offshore environments that are affected by harsh winter conditions where corrosion and ice adhesions are major concerns [64].

Zinc oxide nanostructures were electrodeposited from an aqueous solution at varying salt concentrations and applied potentials [65]. When the concentration of electrolyte was high (5 mM ZnCl_2_ and Zn(NO_3_)_2_), a flake-like microstructure was developed. On the other hand, clusters of nanorods arranged in a flower-like morphology were developed when the concentration of the electrolyte was decreased (0.2 mM ZnCl_2_ and Zn(NO_3_)_2_). Upon surface treatment with stearic acid, the ZnO with hierarchical nanorod/flower morphology became superhydrophobic with a water contact angle of 170°.

#### 3.2.4. Cobalt

Electrodeposited cobalt surfaces with a variety of surface structures were also made superhydrophobic with low surface energy chemical treatment [66,67]. In Li and Kang’s study [66], superhydrophobic cobalt surfaces were deposited on magnesium substrates via a multi-step process. First, electroless nickel-phosphorous (Ni-P) was coated onto the magnesium (Mg) substrate. Second, cobalt was electrodeposited on the Ni-P-coated Mg substrate. Lastly, the electrodeposited cobalt samples were immersed in stearic acid to obtain superhydrophobic surfaces. The superhydrophobic surfaces showed a cotton-like and sharp faceted morphology. After modification with stearic acid, contact angles up to 156° and sliding angles of 1° were observed. The superhydrophobic surface was also exposed to extreme pH and abrasion tests. Except for pH 1–2, the contact angle remained above 150°. An abrasion test was performed by applying a pressure of 1500 Pa on an 800-grit sand paper, and changes in wetting properties were measured as a function of abrasion length. The water contact angle of the Co surface after a 900-mm abrasion length remained above 150°, while the sliding angle increased to 32°. The improved properties obtained by Co electrodeposition and modification can potentially broaden the applications of magnesium alloys [66].

Xiao *et al.* [67] also produced superhydrophobic cobalt surfaces by electrodeposition from aqueous solution and post-deposition chemical modification. The effect of current density on surface morphology was studied. At a high current density of 100 mA/cm^2^, the surface morphology was a nanocone array with an average nanocone root diameter of 250 nm and a height of 400 nm. A shell-like morphology was formed when the current density was decreased to 12.5 mA/cm^2^. The average length and height of the shells were 3 µm and 1 µm, respectively. A hierarchical nanocone-shell structure was also prepared by plating at 12.5 mA/cm^2^ for 20 min first, followed by 100 mA/cm^2^ for 1 min. By treating the surface with stearic acid, Co with nanocone morphology showed a water contact angle of 154°, but the water adhered to the surface, even when it was tilted by 90°. The shell-like structure achieved a similar contact angle of 156° with a sliding angle of 10°. The hierarchical nanocone-shell structure exhibited the best non-wetting properties, with a water contact angle of 160° and a low sliding angle of 4°. Although the water contact angles for the different morphologies were not significantly different from each other, the sharp tips of the nanocone array may cause pinning of water droplets, a result that is consistent with Chen *et al.*’s study [57] when the cone height of metal deposits was low.

#### 3.2.5. Silver

**Figure 6:**
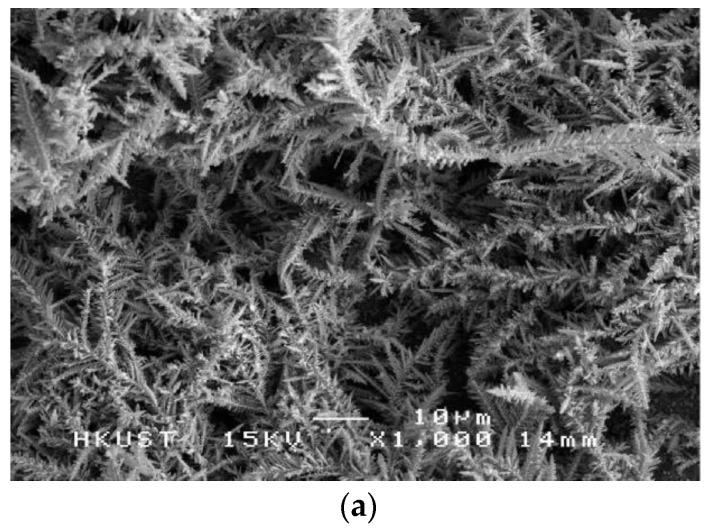

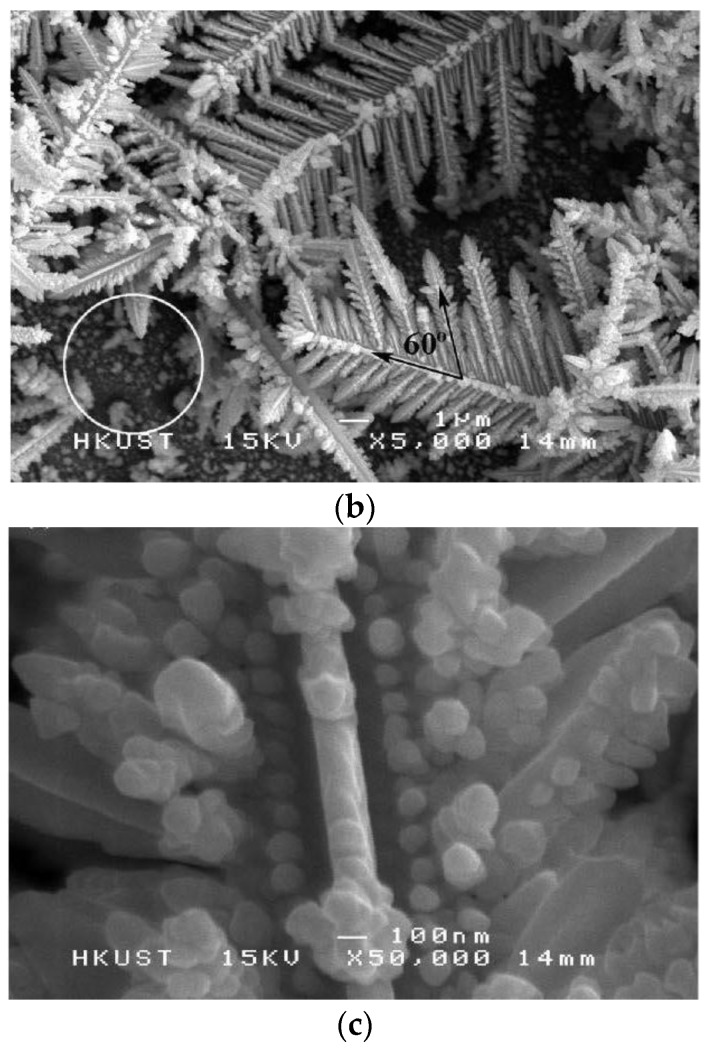
SEM images of the dendritic Ag structure. (**a**) Low magnification; (**b**) medium magnification; (**c**) high magnification. Reprinted (adapted) with permission from [68]. Copyright 2008, American Chemical Society.

Silver surfaces with a dendritic structure were electrodeposited from an aqueous solution of silver nitrate on Ni-coated Cu substrates [68]. It was determined that the applied potential during the electrodeposition of silver had a significant influence on the surface morphology. At applied potentials between −0.4 V and −1.0 V, micron-sized Ag particles with a faceted structure were formed. When the applied potential was −2.0 V, the substrate was covered with a hierarchical structure consisting of micron-sized Ag dendrites and nanoscale Ag crystals on the branches of the dendrites, as depicted in Figure 6. After chemical modification of the dendritic Ag with n-dodecanethiol, a superhydrophobic surface with a water contact angle of 155° and a sliding angle less than 2° was obtained.

#### 3.2.6. Gold

Gold electrodeposits with various surface morphologies were chemically modified to produce superhydrophobic surfaces [69,70,71,72]. In Magagnin *et al.*’s study [69], the effect of current density on the morphology of the gold electrodeposits was examined. At low current density (1 mA/cm^2^), the electrodeposit surface was dense and fairly uniform. With increasing current density, up to 15 mA/cm^2^, aggregated gold particles and dendritic nanostructures were formed. After immersing the gold electrodeposits in n-dodecanethiol, water contact angles up to 179° were reported for a specimen prepared at 15 mA/cm^2^.

In a different study [70], hierarchical dendritic gold structures were electrodeposited on ITO substrates using an aqueous solution. Similar water contact angles (166°) were observed after treatment with n-dodecanethiol. In addition, the coating demonstrated exceptional chemical stability as the water contact angle remained close to 160° for the pH range between one and 13.

Gold surfaces with a cauliflower structure were reported in Ren *et al.*’s work [71]. The deposition process involved two steps, (i) adsorption of gold nanoparticles onto ITO from a colloidal suspension; and (ii) growth of adsorbed particles by electrodeposition in aqueous solution. The as-deposited surfaces showed micron-sized cauliflower structures (5 µm in diameter) that were overlaid with nanoscale gold particles, forming a hierarchical roughness. The as-deposited surfaces were hydrophilic (WCA = 18°). After surface treatment with fluoroalkyl silane, the water contact angle greatly increased to 162°.

pH-responsive, superhydrophobic gold surfaces were prepared by electrodeposition in aqueous solution, followed by a chemical modification in thiols [72]. Clusters of dendritic structure were observed on the as-deposited gold coating. The rough gold surfaces were immersed in a solution of thiols (HS(CH_2_)_9_CH_3_, HS(CH_2_)_10_COOH) to form a mixed monolayer containing alkyl and carboxylic acid groups on the gold surface. pH-responsive wetting behavior was achieved due to deprotonation of the carboxylic acid group on the gold surface. When droplets with a pH between one and seven were placed on the modified surface, the contact angles were above 150°. Between pH 7 and 13, the contact angle decreased significantly, and the surface became superhydrophilic above pH 13; the droplets spread, and the contact angle was close to 0°. After rinsing the base contaminated surface with distilled water, the pH-responsive property was recovered, and the superhydrophobic properties at low pH were again observed. The pH-responsive property of the coating can have important applications in microfluidic switches and controllable separation systems [72].

#### 3.2.7. Palladium

**Figure 7:**
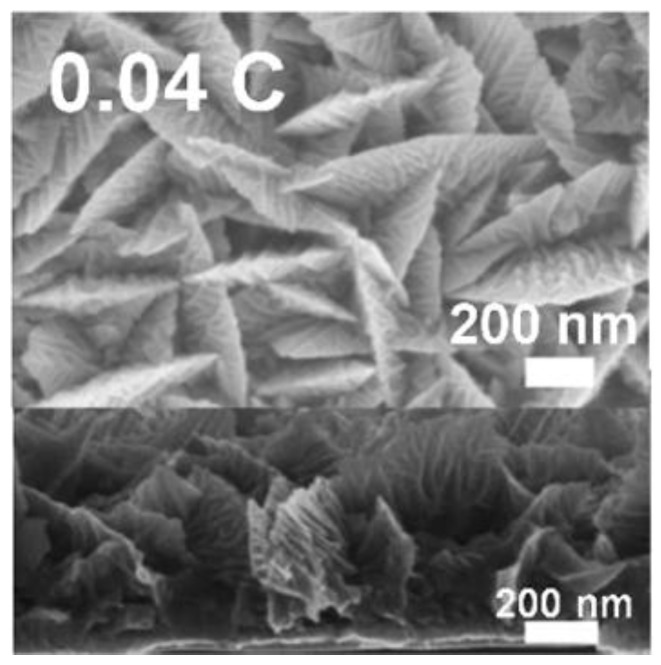
Planar (top) and cross-section (lower) images of the Pd flake structure produced at 0.20 V *vs.* Ag/AgCl and a deposition charge of 0.04 C. Reprinted (adapted) with permission from [73]. Copyright 2015, American Chemical Society.

Superhydrophobic nanoflake palladium surfaces were made by electrodeposition, followed by post-deposition chemical modification with n-dodecanethiol [73]. The authors studied the effect of applied potential and deposition charge on the Pd surface morphology. With increasing applied potential changes in surface morphology were reported from spike-type structures (−0.3 to −0.15 V *vs.* Ag/AgCl) that grew vertically to flake-like nanostructures (0.20–0.25 V *vs.* Ag/AgCl). As shown in Figure 7, Pd deposited at 0.20 V *vs.* Ag/AgCl and a total deposition charge of 0.04 C had a flake-like structure. Before surface treatment with n-dodecanethiol, the water contact angle of the nanoflake Pd surfaces was 60°. After the treatment, water contact angles up to 161° and a sliding angle as low as 3° were observed for a deposition charge of 0.04 C.

#### 3.2.8. Platinum

Superhydrophobic platinum nanowire surfaces were prepared by electrodeposition of platinum nanowire arrays into the pores of anodized aluminum oxide templates, followed by removal of the template and chemical modification of the nanowire array with fluoroalkyl silane [74]. After the removal of the anodized aluminum oxide template, uniform, free-standing Pt nanowire arrays with a hill and valley hierarchical structure were obtained. Each hill (1 µm diameter) was composed of bundles of nanowires (30 nm diameter, 1 µm in height). Before fluorination, the Pt surface showed a hydrophilic behavior, where water droplets spread on the surface. After treatment with fluoroalkyl silane, the water contact angle was 158°, and the sliding angle was less than 3°.

#### 3.2.9. Ni-Cu Alloy

Microstructured Ni-Cu alloy surfaces were electrodeposited from an aqueous solution of dissolved nickel and copper salts, as well as a boric acid as the pH buffer [75]. The morphology of the coating developed from this process was clusters of spherical particles with protuberances. The formation mechanism of the microstructure was similar to the mechanism reported by Yu *et al.* [56] for Ni-Cu-P alloys. Due to copper being a more noble metal than Ni, Cu nucleates on the cathode as spherical particles and is then encapsulated by Ni, which results in spherical to protuberance structures depending on the Cu ion concentration and plating potential. When the alloy surfaces were modified with fluorocarbon, water contact angles up to 158° were achieved.

#### 3.2.10. Cu-Zn Alloy

Superhydrophobic Cu-Zn alloy coatings on magnesium alloy substrates were produced in a multi-step process [76]. First, a Ni coating was deposited on the magnesium alloy substrate by electroless deposition. In the second step, the Cu-Zn alloy was electrodeposited on the Ni-coated Mg substrate. Then, a rough CuO film was developed on the electrodeposited Cu-Zn alloy coating by an anodic treatment. Lastly, the CuO film on the Cu-Zn coating was immersed in lauric acid solution to obtain a superhydrophobic surface. Rough, multi-scale feather-like surface structures were developed from the anodic treatment, providing a structure for air trapping, which led to a water contact angle of 155° and a sliding angle of 3° after modification with lauric acid. A scribe-grid test was performed to evaluate the adhesion property of the deposited film on the substrate. No delamination or detachment of the film was observed, which indicates that the superhydrophobic coating had good adhesion to the substrate.

#### 3.2.11. Zn-Co Alloy

Non-wetting Zn-Co coating was fabricated by electrodeposition from choline chloride-based ionic liquid and surface modification with stearic acid [77]. The Zn-Co coating was composed of nano-sized particles in clusters, forming micron-sized particulates. Before modification with stearic acid, the water contact angle was 0°. After modification, the surface became superhydrophobic (WCA = 152°). The long-term stability of the superhydrophobic property was evaluated by immersion in water for an extended period of time. After 30 h, the water contact angle decreased slightly, but remained above 150°. Extended immersion caused the water contact angle to decrease to 140°, but no further changes were observed beyond 80 h.

### 3.3. Co-Deposition of Second Phase Particles

Co-deposition of hard second phase particles (e.g., carbides, oxides) with a metal is a widely employed electrodeposition technique to produce metal matrix composite coatings for improved properties, such as hardness, strength and wear resistance (e.g., [84,85]). Wetting properties can also be modified by co-depositing inert particles with metal (Table 1, Electrodeposit Category c). In this technique, the electrolyte is an aqueous solution containing dissolved metal salt and inert particles, and sometimes, surfactants are also added to disperse the inert particles. There are two approaches in obtaining non-wetting metal composite coatings by co-deposition: (i) co-depositing inert particles, such as TiO_2_, with metal to achieve hierarchal roughness, followed by surface modification with low surface energy material [78,79,80]; and (ii) co-depositing hydrophobic polytetrafluoroethylene (PTFE) particles with metal [81,82,83].

#### 3.3.1. Ni-TiO_2_ Composite

As documented in three different studies, Ni-TiO_2_ composite electrodeposits can be made superhydrophobic by surface treatment with low surface energy material after electrodeposition [78,79,80]. In the work by Hu *et al.* and Huang *et al.* [78,79], TiO_2_ nanoparticles with a diameter range between 15 and 30 nm were co-deposited with Ni from aqueous electrolyte containing a surfactant. The effect of nanoparticles on the surface morphology and wetting behavior were investigated. A pure nickel coating showed a relatively smooth surface, and after modification with fluoroalkyl silane, the water contact angle of the surface was 131°. When TiO_2_ was added to the plating electrolyte, the resulting composite coating showed a thorn-like morphology with hierarchical roughness, and the water contact angle of the surface increased to 175° after modification. The maximum incorporation of TiO_2_ in the composite was 5 wt %. The authors suggested that when nanoparticles are co-deposited with the nickel matrix, there is a higher density of nucleation sites for nickel ion reduction, and crystal growth is reduced during electrodeposition, leading to a rough morphology that is required for superhydrophobic surfaces.

#### 3.3.2. Ni-PTFE Composites

Several studies looked at the effect of PTFE particles on the wetting characteristics in Ni electrodeposits [81,82,83], all producing contact angles above 150°. For example, Wang *et al.* [82] electrodeposited Ni-PTFE composite coating from a Watts bath containing cationic fluorosurfactant and 0.3-µm PTFE particles. The PTFE content in the composite coating was dependent on the particle concentration in the plating electrolyte. PTFE particles were homogenously distributed throughout the thickness of the composite coating. Increases in surface roughness were observed when PTFE particles were co-deposited with Ni. The maximum PTFE content in the composite was 47 vol % PTFE, and the corresponding water contact angle was 155°. However, no results were presented for contact angle hysteresis or sliding angle.

## 4. More Recent Developments

A demonstrated in Section 3, many different metals have been produced by electrodeposition as superhydrophobic coatings with and without subsequent chemical surface treatment with an intrinsically hydrophobic substance. One of the most critical issues for any superhydrophobic surface is its long-term stability in service for components that are subjected to wear, corrosion and erosion. As long as the surface wetting properties are only controlled by surface morphology and surface treatment with hydrophobic chemicals, the superhydrophobic properties may degrade over time as the top layer is slowly worn away during the service of a coated component. This is one of the biggest technical challenges before such coatings will find full acceptance in large-scale industrial applications. Previous studies that addressed the wear stability will be discussed in Section 5.

**Figure 8:**
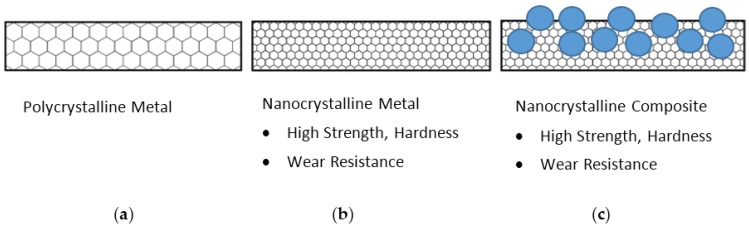
Schematic cross-sectional diagram of various electrodeposits: (**a**) polycrystalline metal; (**b**) nanocrystalline metal with a high density of grain boundaries; (**c**) nanocrystalline metal matrix composite with hydrophobic particles.

We are currently working towards a potential solution to this problem, which is based on the composite coating approach similar to the one described in Section 3.3 that produces a metallic coating with embedded hydrophobic particles throughout its entire thickness, as shown in Figure 3. The concept of this approach is as follows (Figure 8). First, refine the crystal size of the metal matrix from conventional polycrystalline (Figure 8a) to the nanocrystalline range (Figure 8b). By refining the crystal size, electrodeposited metals follow the Hall–Petch relationship of increasing strength σ*_y_* and hardness *H* with decreasing grain size according to the following equations,

(6) $$\sigma_{y}=\sigma_{0}+kd^{-1/2}$$

(7) $$H=H_{0}+k^{'}d^{-1/2}$$

where σ_0_ and *H*_0_ are the strength and hardness at a very large grain size, *k* and *k*’ are constants for each metal and *d* is the grain size [86]. Increasing the hardness of a metal by grain size refinement results in increasing wear resistance, as expected from Archard’s law [87]. The second critical feature of this composite coating is that the hydrophobic second phase particles are uniformly distributed throughout the entire thickness of the coating (Figure 8c). The advantage of this is that new hydrophobic particles will be continually exposed at the surface when the original surface is worn away during service, allowing potentially for long lasting non-wetting surfaces.

### 4.1. Nanocrystalline Ni-PTFE Composites

**Figure 9:**
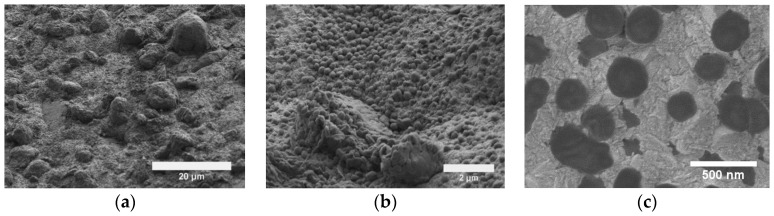
SEM micrographs of the nanocrystalline Ni-PTFE composite showing a lotus leaf-like morphology: (**a**) low magnification; (**b**) medium magnification; (**c**) high magnification.

We have recently developed a superhydrophobic Ni-PTFE with a nanocrystalline Ni matrix [83]. Secondary scanning electron micrographs of such a coating are shown in Figure 9. Dual scale surface roughness with a lotus leaf-like morphology was achieved by co-depositing PTFE powder with a bimodal particle size distribution: irregularly-shaped micron-sized particles with an average particle size of 6 µm and more spherical submicron particles with an average particle size of 0.3 µm. In the low magnification micrograph (Figure 9a), micro-scale protrusions of PTFE particles can be clearly observed. At higher magnification (Figure 9b), it can be seen that the larger PTFE particles are composed of clusters of submicron particles, and the individual submicron particles are uniformly distributed. Nickel matrix, with a very fine grain structure, can be visibly seen growing around PTFE particles in the high magnification image (Figure 9c).

**Figure 10:**
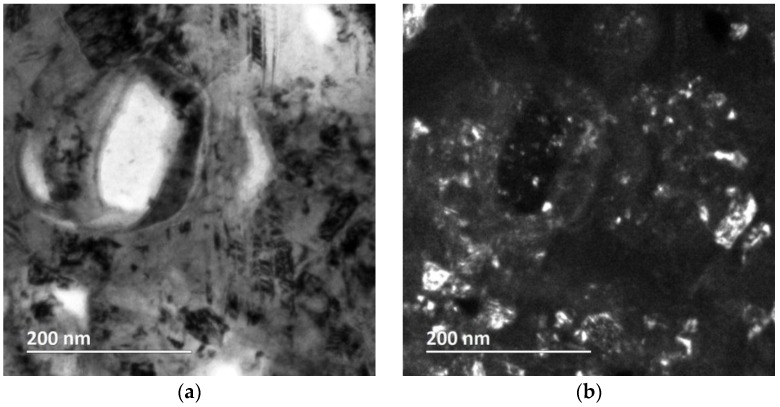

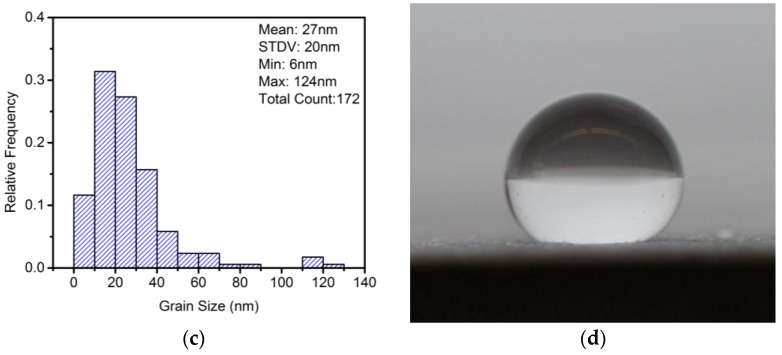
Cross-sectional TEM characterization of nanocrystalline Ni-PTFE composite: (**a**) bright field image; (**b**) dark field image; (**c**) Ni grain size distribution; (**d**) a 5-µL water droplet at rest on nanocrystalline Ni- 70 vol % PTFE coating. Contact angle = 152°.

The grain size of the nanocrystalline Ni matrix was determined with transmission electron microscopy. Crystals with a grain size less than 100 nm can be clearly observed on the cross-sectional bright field and dark field images (Figure 10a,b). The bright area of about 150 nm in diameter seen in the bright field image is a crater that was generated when a PTFE particle was preferentially sputtered out during the focused ion beam (FIB) sample thinning process. The grain size distribution presented in Figure 10c shows an average nickel matrix grain size of 27 nm with a lognormal distribution, which is anticipated for nanocrystalline Ni electrodeposits [86]. The fine grain structure is due to an increase in the effective current density when PTFE particles are co-deposited. Furthermore, the CTAB surfactant used in the bath electrolyte to disperse the PTFE particles is known to have grain refinement properties [88].

The water contact angle of a composite coating containing 70 vol % PTFE was 152° (Figure 10d), and the sliding angle was 30°. This observation suggests that the water droplet is in a mixed Wenzel/Cassie–Baxter state, as the dual scale roughness allows for some trapped air underneath the water droplet, which leads to high mobility of the droplet when the surface is tilted.

**Figure 11:**
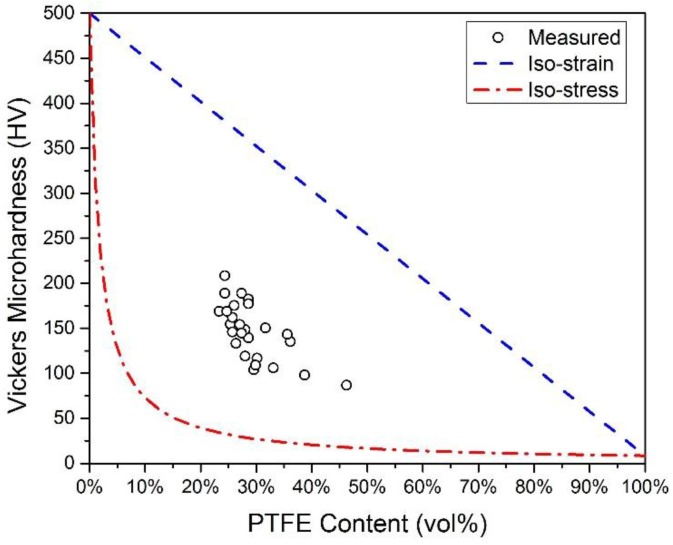
Effect of PTFE on the Vickers microhardness of Ni-PTFE composite coatings.

The microhardness of the Ni-PTFE composite coatings was also evaluated and is shown in Figure 11. It can be observed that the microhardness is strongly dependent on the PTFE content. The iso-strain and iso-stress lines on the plot represent the theoretical upper and lower bound microhardness values according to the rule of mixture composite model [89]. The theoretical limit of 500 HV for Ni without PTFE particles is the microhardness of nanocrystalline nickel with an average grain size of 27 nm [86], and 8 HV for PTFE is based on the estimation made in the previous work [83]. The measured microhardness is between the extreme limits and implies that the composites are in a combination of iso-strain and iso-stress conditions, which is expected for a particulate composite [89].

### 4.2. Rare Earth Oxide as Hydrophobic Particles

Azimi *et al.* [90] recently reported that oxides of the lanthanide series are intrinsically hydrophobic (98° ≤ θ*_Y_* ≤ 115°) due to their electronic structure. Owing to the unfilled inner 4*f* orbitals of the metal atoms that are shielded from interactions with the surrounding environment by a full octet outer shell 5*s*^2^*p*^6^, rare earth oxides have a lower tendency to form hydrogen bonds with water molecules, resulting in hydrophobicity. Furthermore, Azimi *et al.* demonstrated the thermal and mechanical robustness of the ceramics by exposing them to temperatures up to 1000 °C and abrasive wear. They reported that the water contact angle remained about the same after the tests.

**Figure 12:**
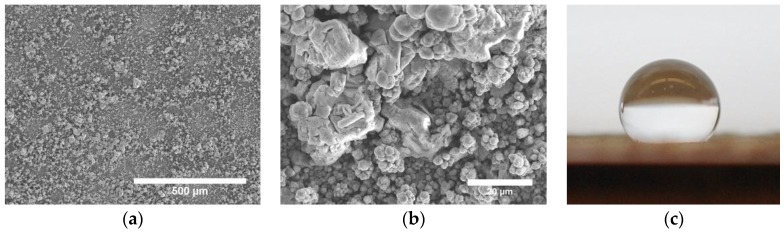
SEM micrographs of Ni-CeO_2_ composite: (**a**) low magnification; (**b**) high magnification image showing aggregates of micron-sized CeO_2_ particles and Ni matrix with a cauliflower-like structure; (**c**) a 5-µL water droplet at rest on a Ni-58 vol % CeO_2_ composite coating. Contact angle = 140°.

The outstanding properties of rare earth oxides make them excellent candidate hydrophobic particles for co-deposition to produce hard and durable superhydrophobic electrodeposits. As a proof of concept, we have co-deposited cerium oxide with nickel and evaluated the wetting properties. Scanning electron micrographs of one of the coatings are shown in Figure 12. A rough surface can be observed in the low magnification micrograph (Figure 12a). In the high magnification micrograph (Figure 12b), agglomerates of micron-sized CeO_2_ particles can be seen surrounded by nickel matrix with a cauliflower-like morphology. The combination of hydrophobic ceramic particles and rough surface morphology significantly increased the water contact angle of Ni (75° [83]) to an average water contact angle of 140° for a composite coating containing 58 vol % ceramic particles (Figure 12c).

## 5. Mechanical and Wear Stability

In order for superhydrophobic surfaces to be applied in practical applications, the material must be able to withstand surface wear and degradation during its lifetime in service. Although superhydrophobic electrodeposits are expected to be mechanically durable, there are only a few studies that investigated the effect of wear on the non-wetting properties [55,58,66,77]. The results of these studies, along with selected reports of wear studies on superhydrophobic materials produced by various other synthesis techniques, are summarized in Table 2.

**Figure 13:**
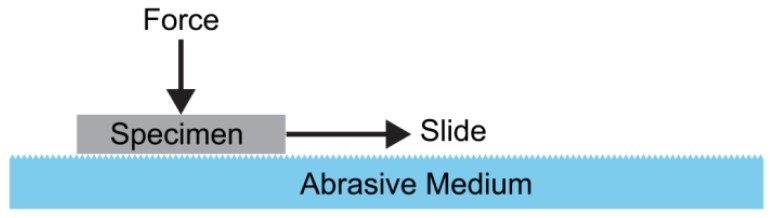
Schematic diagram of a simple abrasion test for superhydrophobic materials.

The most common technique to evaluate the effect of wear on superhydrophobic surfaces is a simple abrasion test (Figure 13): a downward force is applied to the specimen, and it is dragged on an abrasive medium over a set distance. For the superhydrophobic electrodeposits, the abrasive medium common to all reports was abrasive paper. The results showed that electrodeposits remained highly hydrophobic for abrasion lengths up to 1 m.

Other techniques that assess wear on superhydrophobic surface include: (i) the linear abrasion that incorporates a motorized arm with an abrasive attachment that reciprocates on the specimen in a linear motion [101]; and (ii) the falling sand test, in which sand is simply dropped on the superhydrophobic surface from a certain height [99].

Despite the fact that there is a number of studies that evaluate the impact of abrasion on superhydrophobic surfaces, no clear rationales for the experimental parameters were given, such as the coarseness of the abrasive medium and the applied pressure. Unfortunately, no standardized tests are currently used to evaluate the long-term mechanical and wear stability of superhydrophobic surfaces.

## 6. Conclusions and Future Directions

We have demonstrated that there are great numbers of electrodeposition studies to produce metallic superhydrophobic surfaces. The techniques used can be classified into three main categories: (i) rough electrodeposits; (ii) rough electrodeposits modified by low surface energy material; and (iii) composite electrodeposits with hydrophobic particles. The developments of superhydrophobic electrodeposits over the past several years have established the possibility to create non-wetting metal, alloy and composite surfaces from a variety of plating electrolytes. Depending on the deposited metal and specific plating conditions, a very broad range of surface morphologies have been observed, including globular, scaly, spiky, dendritic and spongy structures. On the microscale, deposits were described as leaf-like, flake-like, feather-like, thorn-like, shell-like, flower-like, cotton-like or cauliflower-like in the various studies. It appears that the best non-wetting results were obtained with dual-scale hierarchical structures in which nano-scale features, such as particles, strips, rods, sheets or cones, are superimposed on the micro-sized structure features.

From a commercialization point of view, aqueous electrolytes are likely closest to industrial applications because these solutions are rather inexpensive and easy to control in large-scale plating operations. Today, organic and ionic liquids are: (i) more difficult to handle; and (ii) generally still too expensive, in particular ionic liquids. However, with more research on lower cost approaches to these systems, new commercial opportunities in niche markets could be envisioned, such as smaller-sized microfluidic channels.

As described in the previous sections, electrodeposition from aqueous solutions is a relatively simple and inexpensive technique to produce superhydrophobic surfaces, and the process can be easily scaled up for large surfaces using existing electroplating infrastructure. This could lead to many exciting applications of superhydrophobic metallic electrodeposits. For instance, they can be applied to automotive components or marine structures for reduced corrosion. Superhydrophobic surfaces are also known for anti-icing and delayed ice-formation properties [4,102]. The scalability of aqueous electrodeposition permits it to be a suitable technique to create large superhydrophobic surfaces with anti-icing properties, such as for aircraft parts, wind turbines and power transmission lines and towers for reduced weather-related (e.g., ice storms) operational downtime and structural damage. Pipelines would also benefit from the non-stick properties of superhydrophobic electrodeposits for reduced fluid drag, friction and scale build-up.

Although there has been significant progress in the area of superhydrophobic electrodeposits over the past few years, there remain several issues that need to be addressed before applying this class of materials in industry. As discussed in Section 5, there is a need for a standardized test to evaluate the effect of abrasive and other types of surface wear and degradation on the non-wetting properties. Such tests should mimic the conditions that the surface will be exposed to during service. Another area that should be studied in more detail is the impact of water vapor in air on the hydrophobicity of the surfaces. Studies have shown that when water is condensed on natural superhydrophobic surfaces, such as the lotus leaf, sticky wetting is observed, and the water contact angle decreases due to the presence of condensed water in the valleys of the rough surface structure [103,104]. Therefore, it is critical to develop a suitable technique to evaluate the condensation resistance of superhydrophobic electrodeposits, as the applications described in the various studies are likely under condensation conditions.

## Figures and Tables

**Table 1:** Superhydrophobic electrodeposits, classified into 3 categories: (**a**) Electrodeposits with surface roughness (Section 3.1); (**b**) electrodeposits with surface roughness and chemical modifications (Section 3.2); (**c**) electrodeposits with second phase particles (Section 3.3). WCA, water contact angle; SA, sliding angle.

Electrodeposit Category	Material	Bath Type	Bath Constituents	Voltage/Current Density	Morphology (H: Hierarchical)	WCA (°)	SA (°)	Reference
(a)	Ni	Aqueous	NiCl_2_, H_3_BO_3_, ethylenediamine dihydrochloride	20–50 mA/cm^2^	Nano-micro-cone array (H)	154–156	–	[40,41]
		Ionic	Ethylene glycol, choline chloride, NiCl_2_∙6H_2_O	1.0 V	Nanosheets, nanostrips, cauliflower-like (H)	110–164	3	[42]
		Organic	Ethanol, NiCl_2_∙6H_2_O, myristic acid	30 V	Cauliflower-like (H)	163	2	[43]
	Cu	Aqueous	CuSO_4_, H_2_SO_4_	10–120 mA/cm^2^, 0.1–1.3 V	Lotus leaf-like, cauliflower-like (H)	153–160	8, 5 *	[44,45]
		Organic	Ethanol, myristic acid	5 V	Spiky, flower-like with nanosheets (H)	154	–	[46]
		Organic	Ethanol, nonadecafluorodecanoic acid	10 V	Spiky, flower-like with nanosheets (H)	161	–	[46]
	Co	Aqueous	CoCl_2_, Na_2_SO_4_	−1.0 V *vs.* SCE ^^^	Hierarchical flower-like (H)	162	3.5 *	[47]
		Organic	Ethanol, CoCl_2_, myristic acid	30 V, 20 V	Micro-nano spheres, micro-nanofiber structure (H)	164, 160	2, 6	[48,49]
	Zn	Aqueous	Zn(CH_3_CO_2_)_2_, KCl, NH_4_OH	−1.35 V *vs.* SCE ^^^	Scaly sheets, willow leaf-like with submicron features (H)	170	<1	[50]
	Bi	Aqueous	BiCl_3_, HCl	−1.5 to −2.5 V *vs.* SMSE ^†^	Micron size dendrites with nanoplates (H)	164	–	[51]
	Mn	Organic	Ethanol, MnCl_2_, myristic acid	30 V	Cauliflower-like (H)	163	<3	[52]
	La	Organic	Ethanol, LaCl_3_∙6H_2_O, myristic acid	30 V	Spiky, flower-like with nanorods	165	<2	[53]
	Ce	Organic	Ethanol, CeCl_3_∙6H_2_O, myristic acid	30 V	Spiky, flower-like with interpenetrating network	163	-	[53]
	Ce	Organic	Ethanol, Ce(NO_3_)_3_∙6H_2_O, myristic acid	30 V	Micro-nano papillae (H)	160	1, <2	[54,55]
	Ni-Cu-P alloy	Aqueous	NiSO_4_, CuSO_4_, NaH_2_PO_2_, Na_2_SO_4_, citric acid, sodium dodecyl sulfate	200 mA/cm^2^	Cauliflower-like (H)	153	–	[56]
(b)	Ni + stearic acid	Aqueous	NiCl_2_, H_3_BO_3_, crystal modifier	20 mA/cm^2^	Nanocone array	148–154	0–90	[57]
	Ni + (heptadecafluoro-1,1,2,2-tetrahydrodecyl)-1-triethoxysilane	Aqueous	NiSO_4_, NiCl_2_, H_3_BO_3_	750 mA/cm^2^	Needle-like leaf structure, pine cone-like hierarchical structure (H)	143–162	3	[58]
	Ni + perfluoropolyether	Aqueous	NiSO_4_, NiCl_2_, H_3_BO_3_, saccharin	50 mA/cm^2^	Lotus leaf replica with conical protuberance (H)	156	–	[59]
	Cu + lauric acid	Aqueous	CuSO_4_, KNaC_4_H_4_O_6_, NaOH, H_3_BO_3_	5 mA/cm^2^	Microcone with nanoroughness (H)	154	2	[60]
	Cu + n-dodecanethiol	Aqueous	Cu(NO_3_)_2_	−0.25 V *vs.* Ag/AgCl	Micro spheres with submicron roughness (H)	152	–	[61]
	Cu + fluorocarbon	Aqueous	CuSO_4_, H_2_SO_4_	200 mA/cm^2^	Micro-nano-scale spheres (H)	160	<2	[62]
	Cu + n-octanoic acid	Aqueous	CuSO_4_, H_2_SO_4_	−0.8 to −2.5V *vs.* SCE ^^^	Microclusters with nano-protuberances (H)	153	–	[6]
	Zn + polypropylene	Ionic	choline chloride, urea, thiourea, ZnCl_2_	2.5 mA/cm^2^	Porous, submicron sheet structure	170	–	[63]
	Zn + silicone	Aqueous	ZnCl_2_, Zn(NO_3_)_2_, HNO_3_	−1.4 V *vs.* Ag/AgCl	Micro-nano-fractal morphology (H)	155	2 *	[64]
	ZnO + stearic acid	Aqueous	ZnCl_2_, Zn(NO_3_)_2_, KCl	−0.5 to −1.5 V *vs.* Ag/AgCl	Flower-like with nanorods (H)	170	–	[65]
	Co + stearic acid	Aqueous	CoCl_2_, Na_2_SO_4_	7.5 mA/cm^2^	Hierarchical cotton-like and leaf-like (H)	156	1	[66]
		Aqueous	CoCl_2_, H_3_BO_3_, crystal modifier	12.5 mA/cm^2^, 100 mA/cm^2^	Nanocone array, hierarchical nanocone/shell structure (H)	154–160	4–10	[67]
	Ag + n-dodecanethiol	Aqueous	AgNO_3_	−0.4 to −2 V	Micron size dendrites with nanocrystals (H)	155	<2	[68]
	Au + 1-dodecanethiol	Aqueous	Au_2_S, EDTA, Na_2_SO_3_	1–15 mA/cm^2^	Nanoleaf structure on micro-aggregates (H)	179	–	[69]
	Au + 1-dodecanethiol	Aqueous	HAuCl_4_, Na_2_SO_4_	−0.6 V *vs.* SCE ^^^	Hierarchical dendritic structure (H)	160	–	[70]
	Au + fluoroalkyl silane	Aqueous	HAuCl_4_, polyvinylpyrrolidone	1.0 V	Cauliflower-like (H)	162	–	[71]
	Au + thiols	Aqueous	HAuCl_4_, H_2_SO_4_	−0.2 V *vs.* Ag/AgCl	Dendritic structure with nanobranches (H)	154	–	[72]
	Pd + n-dodecanethiol	Aqueous	K_2_PdCl_4_, H_2_SO_4_	−0.3 to 0.25 V *vs.* Ag/AgCl	Spiky, nanoflake structure (H)	161	3	[73]
	Pt + fluoroalkyl silane	Aqueous	H_2_PtCl_6_, HCl	0 V *vs.* SCE ^^^	Nanowire bundles (H)	158	<3	[74]
	Ni-Cu alloy	Aqueous	Ni(NH_2_SO_3_)_2_, CuSO_4_, H_3_BO_3_	−0.9 to −1.5 V *vs.* Ag/AgCl	Microspheres with nano-protrusions (H)	158	10	[75]
	CuO-Cu-Zn alloy + lauric acid	Aqueous	CuSO_4_, ZnSO_4_, KNaC_4_H_4_O_6_	6 mA/cm^2^	Multi-scale feather-like structure (H)	155	3	[76]
	Zn-Co alloy + stearic acid	Ionic	Choline chloride, urea, ZnCl_2_, CoCl_2_	3.5 mA/cm^2^	Micro- and nano-particles in clusters (H)	152	–	[77]
(c)	Ni-TiO_2_ composite + fluoroalkyl silane	Aqueous	NiSO_4_, NiCl_2_, H_3_BO_3_, sodium dodecyl sulfate, TiO_2_	60 mA/cm^2^	Micro- and nano-particles (H)	152	–	[78]
		Aqueous	NiSO_4_, NiCl_2_, H_3_BO_3_, Polysorbate 80, TiO_2_	14–50 mA/cm^2^	Hierarchical thorn-like structure (H)	175	–	[79]
		Aqueous	Ni(SO_3_NH_2_)_2_, NiCl_2_, H_3_BO_3_, TiO_2_	2.3–54 mA/cm^2^	Nanoparticles in micron size agglomerates (H)	157	–	[80]
	Ni-PTFE ^‡^ composite	Aqueous	Ni(NH_2_SO_3_)_2_, NiCl_2_, H_3_BO_3_, cationic surfactant, PTFE	30 mA/cm^2^	Microscale fractal morphology	156	–	[81]
		Aqueous	NiSO_4_, NiCl_2_, H_3_BO_3_, cationic fluorosurfactant, PTFE	50–100 mA/cm^2^	Submicron roughness	155	–	[82]
		Aqueous	NiSO_4_, NiCl_2_, H_3_BO_3_, cetyltrimethylammonium bromide, PTFE	100 mA/cm^2^	Lotus leaf-like (H)	152	–	[83]

* Contact angle hysteresis; ^^^ SCE: Saturated calomel electrode; ^†^ SMSE: Saturated mercury sulfate electrode; ^‡^ PTFE: Polytetrafluoroethylene.

**Table 2:** Various wear tests of superhydrophobic surfaces.

Wear Test	Superhydrophobic Material (E: Electrodeposit)	Abrasive Medium	Pressure (Pa)	Abrasion Length (mm)	Initial WCA (°)	Final WCA (°)	Initial SA (°)	Final SA (°)	Reference
Simple abrasion	Ce (E)	1000-grit abrasive paper	1300	500	160	148	< 2	–	[55]
	Ni + (heptadecafluoro-1,1,2,2- tetrahydrodecyl)-1-triethoxysilane (E)	800-grit abrasive paper	1200–6000	1000	162	148–159	3	5–31	[58]
	Co + stearic acid (E)	#800 abrasive paper	1500	1100	156	148	1	40	[66]
	Zn-Co alloy + stearic acid (E)	#5 abrasive paper	–	–	152	145	–	–	[77]
	Microstructured PTFE film, 100 µm thick	P1500 abrasive	2700	4500	152	147	11	18	[91]
	UHMWPE ^1^ substrate with silver + fluorinated	1500 mesh abrasive paper	10,000	3000	163	160	5	15	[92]
	Polyester fabric with silver + fluorinated surface	1200 mesh abrasive paper	13,000	–	159	153	5	18	[93]
	Fluorinated silica nanoparticles/TiO_2_ nanocomposite	1500 mesh abrasive paper	20,000	225	155	139	5	70	[94]
	Cotton fabric with structured co-polymer	1000 mesh abrasive paper	3920	8000	158	150	3	18	[95]
	Polydimethylsiloxane elastomer	Abrasive paper	2000	800	165	152	–	–	[96]
	polyvinylidene fluoride PVDF)/fluorinated ethylene propylene/carbon nanofibers composite	1000 mesh abrasive paper	500,000	–	164	141	5	20	[97]
	Copper sulfide film + stearic acid	Cotton fabric	5000	250	152	143	–	–	[98]
	Hierarchical Si + PFOS ^2^	TechniCloth^®^	3450	250	169	167	2	14	[99]
	SiO_2_ nanoparticle/epoxy composite + fluoroalkyl silane	TechniCloth^®^	3450	3000	169	165	2 *	62 *	[100]
Linear Abrasion	Titanium + fluoroacrylic polymer	H-18 0.25′′ Taber abradant	10,800–433,700	–	165	105	7 *	60 *	[101]
Sand Abrasion	Hierarchical Si + PFOS	140 mesh sand	Sand dropped from 30 cm above the specimen	N/A	165	161	1	70	[99]

* Contact angle hysteresis; ^1^ UHMWPE: Ultra high molecular weight polyethylene; ^2^ PFOS: perfluorooctyl trichlorosilane.
